# Paraneoplastic Syndrome in Splenic Marginal Zone Lymphoma: A Rare Phenomenon of Paraplegia as an Atypical Presenting Manifestation

**DOI:** 10.1155/2016/7034167

**Published:** 2016-05-11

**Authors:** Jessica Schering, Vijayalakshmi Donthireddy

**Affiliations:** Hematology/Oncology Department, Henry Ford Hospital, Detroit, MI 48202, USA

## Abstract

We describe a case presenting complaint of complete lower body paraparesis, which was discovered to have splenic marginal zone lymphoma (SMZL). While paraneoplastic syndromes are more common in tumors, such as small cell lung cancer, very few reports exist on this condition with SMZL. We describe such a rare entity with a clinical course spanning twenty-four months after diagnosis.

## 1. Introduction

Splenic marginal zone lymphoma (SMZL) is a rare disease, originating from mature B-lymphocytes that are normally present in the marginal zone of lymphoid follicles found in the lymph nodes, spleen, and mucosal lymphoid tissues [1]. Classified as an indolent lymphoma, it accounts for less than one percent of all NHLs [2].

Presenting clinical features of SMZL include splenomegaly, lymphocytosis, or cytopenias. Unlike other NHLs, lymphadenopathy and involvement of extralymphatic organs are uncommon. Splenic pathologic examination can show follicles with expanded mantle and marginal zones and areas of coalescence [3]. The immunophenotype is characterized by expression of CD20 but lack of CD5 or CD10, which is useful in distinguishing this disease from other indolent lymphomas [4].

Splenectomy was considered the optimal first-line therapy in symptomatic patients. Splenectomy alone can achieve an ORR of 80–90% [5, 6]. With the addition of chemotherapy to splenectomy, complete response rates can be improved, though historically known to be with moderate activity [7]. Rituximab alone or in combination (e.g., bendamustine) has been shown to be effective with overall response rates greater than 90%, with almost half of responses being complete, while the 5-year progression-free survival is approximately 70% [8–12].

Prognosis is generally good with median overall survival of 10 years, though some have a more aggressive course.

## 2. Case Presentation

We present a case of a 64-year-old Caucasian female with no significant medical history, presenting with an ascending bilateral lower extremity weakness and numbness and tingling that started a month prior. She complained of worsening weakness leading to paraplegia with night sweats present for a week prior to presentation. Initial lab work revealed a hemoglobin level of 10.6 g/dL and platelet count of 136 K/*μ*L. Her WBCs were within normal range, although with increased absolute lymphocytes and monocytes.

Initial examination showed 3/5 strength of hip flexors, 4/5 knee flexors, and 5/5 plantar flexors. During inpatient course, neurologic status worsened to a strength of 1/5 in hip flexors and 0/5 strength bilaterally of hip extensors, abductors, adductors, knee flexors, extensors, ankle dorsiflexors, plantar flexors, foot inverters, evertors, toe extensors, and flexors. Her reflexes were 2/4 in the biceps, triceps, and brachioradialis, and the knees bilaterally, however, were 0/4 in the bilateral ankles. A positive Babinski sign was noted bilaterally.

MRI of the brain and complete spine did not disclose any infiltrative process, though diffuse hypointensity within the visualized cervical and thoracic spine was suspicious for diffuse bone marrow replacement process. Electromyography showed mild sensorimotor peripheral neuropathy. Bloodwork showed normal copper levels and paraneoplastic profile (ANNA-1,-2, and -3; AGNA, PCA 1,2, and Tr; Amphiphysin, CRMP-5). Viral studies including HTLV-2, ENA, ACE, and PRP were negative. HIV ELISA was reactive, although western blot was negative. HepBsAg was positive while HepB DNA was negative. IgM cryoglobulins were elevated at 2.0 mg/dL. CSF analysis showed 12 WBCs and lymphocytic pleocytosis with oligoclonal bands. West Nile Virus IgG antibodies were positive, though IgM antibodies were negative. Due to suspicion of Guillain-Barré syndrome, she was initiated on plasmapheresis. She received five treatments with no improvement of her presenting symptoms.

Initial workup for her anemia showed normal iron indices, B12, and folate levels. Rheumatoid factor and ANA were negative. JAK-2 mutation was not present. Reticulocytes were at 1.9%. Bone marrow biopsy was performed. Microscopic evaluation revealed 80% replacement of her marrow with mature B-cell lymphoma (Image 1 in Supplementary Material available online at http://dx.doi.org/10.1155/2016/7034167). Morphology consisted of small mature B-cells that were seen extensively infiltrating the bone marrow (Image 2). Flow cytometry revealed a clonal population of CD5-negative/CD10-negative phenotype. Due to the morphologic nature and CD103 negativity, hairy cell leukemia was deemed less likely. Given the lack of an identifiable M-component on serum protein electrophoresis and the splenomegaly present, lymphoplasmacytic lymphoma was also unlikely. Cytogenetic evaluation revealed the loss of 7q and a gain of chromosome 3 (3 copies of 3q27 BCL6 probe) which is observed in marginal zone lymphoma. On imaging, no intrathoracic, intra-abdominal, retroperitoneal, or pelvic lymph nodes were present. All features above were suggestive of splenic marginal zone lymphoma and treatment was planned.

She was initiated on IV rituximab 375 mg/m^2^ (day 1/28) with bendamustine 90 mg/m^2^ (days 2-3/28) regimen (BR) every twenty-eight days for six cycles. Considering her hepatitis B surface antigen positivity, tenofovir was given prophylactically, although hepatitis B DNA quantitative evaluation was negative with <10 IU/mL.

She tolerated treatment well. After cycle #2, her pancytopenia improved. She was able to flex and extend her toes and knees without improvement in her hip flexors or extensors. After cycle #3, her fatigue and night sweats started to improve and she was able to stand shortly without assistance. She continued to work with physical therapy and at the completion of final sixth cycle of BR, she was able to stand up without assistance and ambulate on her own, nearly six months after her initial presentation.

Repeat bone marrow biopsy done approximately three weeks after her last cycle showed no immunophenotypic evidence of a mature B-lymphoid neoplasm and normal flow cytometry. Complete imaging done three months after therapy showed significant reduction in splenic size from 26 cm to 16 cm with no adenopathy noted. Further surveillance continued to show splenic size reduction to 14 cm.

24 months after diagnosis, the patient continues to enjoy life at home, fully ambulatory and disease-free.

## 3. Discussion

The morphology of SMZL is characterized by an infiltrate of centrocyte-like small cleaved cells, monocytoid B-cells or small lymphocytes with a round nucleus, condensed chromatin, and abundant basophilic cytoplasm with small surface “villous” projections [13]. Splenic hilar lymph nodes are commonly involved, though marginal zone pattern is variable [14]. Bone marrow involvement can be subtle with lymphoid aggregates, variable marginal zone pattern and intrasinusoidal lymphoid infiltration [15]. About 20–25% of patients may be observed to have no cytopenias or symptomatology [16].

There are previously reported causes of neurologic symptomatology including Guillain-Barré syndrome and sarcoidosis in SMZL. Our patient was evaluated for such phenomenon and workup was negative. She was treated empirically with plasmapheresis without improvement. Although there has been a radiographic report published in the literature so far, no clinical documentation of paraneoplastic paraplegia in SMZL has been reported [17]. Moreover, complete resolution of paraplegia after treatment of the underlying SMZL has not been reported.

Being a systemic disease with significant morbidity and mortality, splenectomy alone is being questioned as first-line therapy, with systemic therapy being accepted as the mainstay of treatment. This case exhibits a case of SMZL, accompanied by a rare phenomenon of a paraneoplastic paraplegia which resolved completely with treatment of the lymphoma.

## 4. Conclusion

Splenic marginal zone lymphoma (SMZL) is a rare disease, classified as a mature B-cell indolent lymphoma, accounting for less than one percent of all NHLs. Being a rare disease presenting as an atypical phenomenon of paraplegia, this patient improved with treatment and achieved complete response of disease and paraplegia. This is the first known case of such a rare entity clinically described.

## Supplementary Material

Supplementary material contains bone marrow aspirate and biopsy pathology slides for review.
