# Supplementary material for: Paraneoplastic Syndrome in Splenic Marginal Zone Lymphoma: A Rare Phenomenon of Paraplegia as an Atypical Presenting Manifestation
Source: Case Rep Hematol. 2016 May 11;2016:7034167. doi: 10.1155/2016/7034167 (PMC4879228; doi:10.1155/2016/7034167)
Supplement: Supplementary file 1 — Supplementary material contains bone marrow aspirate and biopsy pathology slides for review. [file 7034167.f1.pdf]

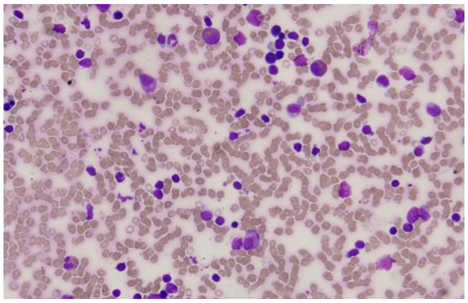

Image 1.

Image 1: Bone marrow aspirate showing lymphocytes with abundant cytoplasm.

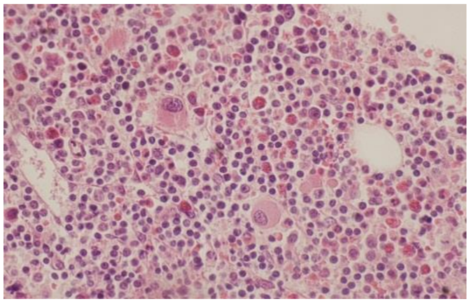

Image 2.

Image 2: Bone marrow biopsy showing a hypercellular marrow primarily occupied by the extensive lymphoid infiltrate composed mostly of small mature lymphocytes.
